# Obstetrics and Gynecology

**Published:** 1894-09

**Authors:** 


### OBSTETRICS AND GYNECOLOGY.

EDITED BY DR. VIRGIL O. HARDON.

### TREATMENT OF VAGINITIS.

Lutaud (*Revue Obstétricale et Gynécologique,* January, 1894). The treatment of vaginitis differs little, whether it be simple or specific. The physician should, above all, consider the *acute* and *chronic* vaginitis in the therapeutical point of view.

(*a*) *Acute Stage.*—Here the speculum should not be employed. The patient should be kept as quiet as possible, and walking, coitus, and all physical exercise forbidden. Frequently repeated injections (every six hours) with Esmarch's douche with two litres of a one per cent. solution of boric acid. Emollient injections, such as starch, flax-seed meal or decoction of poppy heads, may also be employed. If pain is severe, one of the following suppositories is given every evening:

℞ Ext. opii.......................... .................... ........................centigm. i.

Ol. theobrom..... ...................... .................. ..... ..........gms. iv.

M. Ft. supposit. No. 1.

This may be replaced by a little injection containing fifteen drops of laudanum. For bladder symptoms (tenesmus, pain during micturition), poultices with laudanum sprinkled over them, bromide of potassium, emollient drinks, and alkaline diuretics are to be prescribed. It is well in these cases to discontinue wine, but light tea may be taken.

(*b*) *Chronic Stage.*—An injection three times a day is to be given with one of the following:

℞ Acid carbol................ ..................... ..... .............. .......gms. v.
Alcoholis...................... ....... ...... ..................... ......gms. x.
Ess. thym......... ... ....... ........................... ...........gtts. xx.
M. For two quarts of water.

℞ Hydrarg. bichlor...... .................. .. ...............centigms. xxv.
Acid. tartaric ....................................... ...................gm. i.
M. For two litres of water.

℞ Potass. permanganat.................. ................ .................gms. x.
Ac. dest...................................... ............... ...................gms. cc.
M. A tablespoonful for two quarts of water.

This last is the best remedy for gonorrheal vaginitis; if it did not soil linen, it would be perfect. In rebellious cases, or when it is necessary to act rapidly, a Sims speculum is introduced and the vagina is painted with a solution of nitrate of silver (two grammes in thirty grammes of water).

Before withdrawing the speculum, a large tampon imbibed with the following is introduced:

| | | |
|---|---|---|
| ℞ | Acid. tannic | gms. ii. |
| | Cocain. hydrochlorat | centigms. x. |
| | Glycerini | gms. cxx. |

The tampon should be left in place for two days. No matter what may be the treatment applied in chronic vaginitis, the cure may always be hastened by the vaginal dressings, which isolate the parts and absorb the secretions. Simple dry cotton tampons are good, but when made with iodoform or salol gauze are preferable.—*Annals of Gynecology*.

---

### Albuminuria in Pregnancy as a Cause of Death to the Fetus.

Dr. Oui (*Journal de Médecine de Bordeaux*, October 15, 1893), since last January, has treated twelve cases of albuminuria in pregnant women; eight of the children died, four of them lived. Of the eight deaths, one was due to hemorrhage from a bad insertion of the placenta; another from hemorrhage from the meninges. There remain six fatal cases out of twelve, consequently fifty per cent., in which four were found to have mutiple intraplacental hemorrhages. These hemorrhages are frequently met with in albuminuria of pregnancy, and many attain very great proportions. Dr. Oui cites a case in which the clots adherent to the placenta weighed as much as the organ itself. It is to be remarked that these were found, with but one exception, in women with albuminuria, who had neglected themselves, and had not been put on milk diet. Consequently, it is not only for the mother, but also for the fetus, that the milk diet is very useful, and this is shown in a recent statistic of Bridier, relating to 250 cases of albuminuria, the mortality for the children being only twenty per cent., as the greater number of the women had been treated, making a great contrast with the author's cases, where the mothers had been without treatment. Bringing about premature labor before the death of the child is a conduct to be discussed, but this may be done, if, after a milk diet, no amelioration in the albuminuria is produced. On *résumé*, absolute milk diet, and if the albuminuria does not diminish, then induce premature labor.—*Revue Générale de Médecine*, December 6, 1893.

---

### PRECAUTIONS NECESSARY IN THE USE OF PESSARIES.

Neugerbauer has collected 282 cases in which pessaries have been the cause of more or less grave lesions of the genital organs, including twenty-three perforations of the bladder, twenty of the rectum, one of both the bladder and rectum, two of the *cul-de-sac* of Douglas, three of penetration of the pessary into the pelvic connective tissue, six into the uterine cavity, four of introduction of the pessary into the bladder through a fistula, and a very large number of cases of para and peri-metritis and even peritonitis and septicemia, including eight fatal cases, eight cases of carcinoma of the vagina, and many cases of incarceration in which the pessary could be extracted only by surgical means.

In spite of this long and varied list of accidents the author by no means rejects the pessary from the gynecological armamentarium, but does insist that when the use of a pessary is decided upon great care shall be taken as to its form, size, and the substance from which it is made, and that furthermore, the patient shall subsequently be under the control of the physician. N. himself generally uses globular or ovoid pessaries.

The author lays great stress on the point that the patient should exactly understand how to withdraw the pessary herself. In cases of prolapsus the patient should understand how to withdraw the pessary at night and insert it in the morning herself, and in cases of pessaries used for retro-displacements the patient should be taught to withdraw the pessary herself on feeling the slightest pain from its presence, and not be forced to wait until her physician can do it for her.—*Ex.*

### THE MIGRATION OF THE OVUM TO THE TUBE.

Lode has investigated this interesting subject by a series of experiments upon guinea-pigs. In one experiment he injected fine coal particles into the peritoneal cavity. The animal was killed thirty-six hours afterward and the coal particles were detected in both tubes. In a guinea-pig four months old, in which the sexual organs were not yet fully developed, the particles were found only in the fimbria. In another series of experiments he used the ova of the ascaris lumbricoides, as they were larger than the coal particles. The injections were made in the same way into the peritoneal cavity. The examinations of the guinea-pigs killed within a variable period of between thirty-six hours to seven days showed the presence of the ova in large numbers in about the middle portion of the tubes. In a few instances the ova had become glued together, forming a round mass fully the size of the natural ovum of the guinea-pig. This was found in the same situation in the tubal canal. The author draws the following conclusions from his investigations.

1. The cilia of the tube of a guinea-pig have the power of setting into motion bodies the size of the ovum of the guinea-pig, providing that the animal has arrived at sexual maturity.—*Archiv für Gynäkologie,* Bd. xlv. Heft 2.

---

### THE DANGERS IN ANTISEPTIC MIDWIFERY.

Schrader has called attention to the exceptions in the success of antiseptic obstetrical practice, and endeavors to explain the cause why the practice is not universally successful. He holds that the poison of puerperal endometritis is often actually diffused throughout the body by intra-uterine irrigations, thus causing a local and probably a harmless morbid condition to become the agent of deadly infection, the veins and lymphatics acting as the diffusers. The stimulation of the uterine muscular tissue by the injections increases the lymphatic absorption. Although vaginal injections are much safer, he believes that they frequently cause a general infection. The fact that a streptococcus has been found to be normally present in the vaginal secretions after labor by Doderlin is considered of importance. Schrader strongly condemns the use of caustics in puerperal ulcers. The reason for the rapid healing of a lacerated perineum when it is repaired immediately after labor is that the parts are allowed to rest. Glockner's statistics are not favorable to prophylactic irrigation.

---

### A Simple Method of Plugging the Vagina.

Guéniot remarks that applying tampons to the vagina is sometimes painful to the patient and difficult for the physician. He is in the habit of using for this purpose a narrow instrument three and a half inches long and about one wide. It is bent at a right angle, so that the hand holding it shall not interfere with the other hand introducing the tampon. The right hand with this instrument raises the anterior vaginal wall. The posterior wall being then pressed down by the physician's left forefinger, sufficient space is left for the easy application of the tampon. This method is especially applicable to the use of gauze, the introduction of which, without some widening of the vagina, is apt to be painful.—*Ex.*

